# User Testing an mHealth Behavioral Health App for Hopi/Tewa Youth During the COVID-19 Pandemic: Usability Study

**DOI:** 10.2196/77898

**Published:** 2026-03-24

**Authors:** Shelby Hagemann, Morgan Vigil-Hayes, Ashish Amresh, Marissa Adams, Darold H Joseph, Ann Futterman Collier

**Affiliations:** 1 Northern Arizona University, S San Francisco St, Flagstaff, AZ, 86011, United States, 1 928-523-0101; 2 Michigan State University, East Lansing, MI, United States; 3 Colorado School of Public Health, Aurora, CO, United States

**Keywords:** mobile app, mHealth, mobile health, mental health, Native American, American Indian/Alaska Native, remote user testing

## Abstract

**Background:**

American Indian/Alaska Native (AI/AN) people represent a culturally diverse people group within the United States. AI/AN people experience some of the most severe health disparities in the United States, including behavioral health. A quarter of AI/AN people in the United States live on tribal lands, experiencing significant barriers to mental health resources and broadband infrastructure for telehealth. We developed Amplifying Resilience Over Restricted Internet Access (ARORA)—a mobile health (mHealth) smartphone app, promoting mindfulness practices and community building through AI/AN culture and values. Originally co-designed with both Hopi/Tewa and Navajo youth and adults, this study evaluated app resonance among Hopi/Tewa youth, supporting its iterative design. While we initially planned in-person user testing, this was moved online due to the COVID-19 pandemic.

**Objective:**

This study assessed the potential and acceptability of an mHealth app supporting Hopi/Tewa youth practicing mindfulness inspired by their culture, values, and beliefs. This research served as preliminary work for an ongoing, iterative participatory action research study, identifying points of improvement to align with our partner community’s goals.

**Methods:**

After meeting with 6 community advisory board members and focus groups prior to this study, we developed a prototype for ARORA. This study evaluated intuitiveness and usability through testing and interviews with Hopi/Tewa youth. All meetings with stakeholders were moved online due to the COVID-19 pandemic. Using screen-sharing via Zoom (Zoom Communications, Inc) and Android emulators, we received feedback for the iterative design process.

**Results:**

This study involved 9 participants aged 16-24 years. Of these participants, 1 was male and 8 were female; all identified as Hopi/Tewa and/or Tewa. This study included a quantitative assessment using a modified version of the User Version of the Mobile Application Rating Scale. The mean score across all questions was 3.71 (SD 0.427), suggesting generally positive reception. Qualitative results from thematically analyzing open-ended focus group data produced 5 open codes and 12 axial themes, reaching thematic saturation after engaging with 9 participants. Qualitative feedback revealed that while its use was generally enjoyable, the ARORA app could be more specific to Hopi/Tewa culture. Finally, we reflect on adaptations made to our initial protocol in response to the COVID-19 pandemic, offering guidelines for future mHealth work involving rural or hard-to-reach communities.

**Conclusions:**

In this evaluation and usability testing of the ARORA prototype, participants expressed interest and engagement in the mindfulness activities. Participants also identified spaces in which the app could improve, both in usability and in cultural groundedness, especially with the visual dimensions of the app. Reflecting on our experience in facilitating remote user testing, we encourage future work in rural mHealth to consider practices for conducting research when in-person meetings are not feasible.

## Introduction

American Indian/Alaska Native (AI/AN) people represent a culturally diverse group within the United States, with 574 federally recognized Indigenous tribal communities [1], each with their own distinct culture. AI/AN people experience some of the most severe behavioral health disparities in the United States, with suicide rates that are 2.1‐4.1 times higher for youth (aged 14‐24 years) than for White counterparts [2], and approximately 60% of AI/AN youth experiencing severe mental health distress [3]. Of the 9.7 million AI/AN people living in the United States, 25% live on tribal lands [4], which have very limited access to mental health resources [5] and secure broadband infrastructure, which is needed for supporting telehealth [6]. Moreover, there is often a lack of culturally centered mental health interventions for AI/AN communities [7].

Mobile health (mHealth) addresses mental and behavioral health care gaps among rural communities [8]. However, the design of mHealth technologies for hard-to-reach communities must be informed by the target populations’ infrastructural affordances. Mental health and wellness practices operate most effectively when they are tailored to specific communities and populations [9], presenting challenges for tribal communities, who historically have not been included in the technology design process [10].

mHealth supports technologies designed for health care, with broad applications in the mental and behavioral health spaces [11-14]. mHealth has the potential to address barriers to care, resulting from geographical restraints, by supporting remote interventions. Prior work has highlighted mHealth’s capabilities for addressing hard-to-reach populations, such as rural communities [15,16], older adult populations [17-19], and Indigenous populations [20]. Many works, particularly in the human-computer interaction research space, have noted the influence of relationality, identity, and culture in mHealth [21-23]. Universalist interventions fail to account for the identity-based contexts that inform user health and wellness, such as historical trauma or culturally specific familial dynamics [21-23]. Our work with the Hopi/Tewa community differed from prior Indigenous mHealth work in that it centers on a specific population underrepresented in the current literature. In the United States alone, there are 574 federally recognized Indigenous communities, each with their own distinct cultures, histories, and values, meaning that existing mHealth technologies for AI/AN communities may not resonate with all Indigenous communities.

Our work prioritized designing for rurality, as Hopi land is rural. An important factor in developing mHealth for rurality is understanding the target community’s affordances. Wyche et al [24] deployed an mHealth app for monitoring diabetes among youth in Kenya, noting the challenges and limitations of developing a smartphone-based tool in rural areas with limited internet and electricity access. The authors’ contributions support growing arguments for mHealth research to design for existing capacities at community scale, recognizing what tools are readily available for stakeholders and what cultures surround them [24,25]. Okumu et al [15] use mHealth to offer Sexual Reproductive Health information to displaced populations in Uganda, noting the importance of developing digital health content grounded in local cultures. Indeed, intersectionality plays a significant role in designing mHealth for rural communities, as they not only have distinct identity-based needs but also often overlap with culturally distinct groups. We adopted valuable guidelines from prior participatory action research (PAR), informing this work with the network issues existing on Hopi/Tewa land.

We acknowledged known challenges and needs for developing mHealth for rural communities, further tailoring our work to cater toward the remote Hopi/Tewa community. We designed Amplifying Resilience Over Restricted Internet Access (ARORA)—an mHealth smartphone app that incorporates Southwestern AI/AN culture and values into its design while promoting mindfulness practices and community building. Initially co-designed with Hopi/Tewa and Navajo community advisors and AI/AN youth in 2019 and 2020 [26], we developed a functional prototype of ARORA in 2020, while simultaneously solidifying partnerships with Hopi/Tewa Tribe Behavioral Health Services. This study reviewed the iterative and ongoing design of ARORA, requesting early feedback from stakeholders on the functional prototype, to identify needs for improvement. In particular, we reviewed the acceptability and usability of the app. In preparing for this trial, we did not publish a protocol, as guided by our National Science Foundation–funded grant proposal. Rather, we followed a PAR approach [27], maintaining adaptability both in the protocol and the design of the prototype. PAR prioritizes adaptability. We aimed to meet the needs of our target population, building trust and developing a digital artifact that resonates with the stakeholders.

Due to the COVID-19 pandemic, we had to adapt our research protocol to accommodate remote app evaluations. Initially, user testing sessions were planned to take place on Northern Arizona University’s campus, with groups testing ARORA together. Rather than ceasing research due to our inability to meet in-person with participants, we pivoted to holding user testing sessions over Zoom (Zoom Communications, Inc). These evaluations offered valuable community-based feedback to be used in informing iterative versions of ARORA. Pandemic-informed adaptations to the protocol allowed us to review how users would engage with ARORA in the contexts that it ultimately would be used in: on Hopi/Tewa land and in the homes of youth.

Evaluating an early prototype in context, allowing for feedback to inform its iterative design, aligns with design concepts, such as Formative Situations. This design philosophy entails reviewing how stakeholders engage with technology within a variety of scenarios, prioritizing consistent and iterative feedback [28]. Remote user testing presented challenges that are necessary for understanding ARORA’s iterative design, such as intermittent internet connectivity. Challenged network and internet access is common in rural spaces in the United States, including on Hopi/Tewa land [29]. Holding user testing sessions remotely highlighted unique needs for further adapting ARORA to operate effectively under these circumstances.

Our previous work prioritized designing ARORA, determining the activities, aesthetics, and language that were to be included in it [26]. While initial work on this project involved some user testing, ARORA was initially designed for Southwest youth in more broad contexts, rather than being Hopi/Tewa-specific. Therefore, in this work, we aimed to address the following questions:

- *Research question 1:* How can ARORA be adapted to better resonate with Hopi/Tewa culture, thus supporting Hopi/Tewa youth in practicing emotional wellness and resilience?
- *Research question 2:* Does ARORA feasibly support youth in engaging with emotional resilience?

The contributions of this study are as follows:

- We conducted a remote user study with 9 Hopi/Tewa youth (aged 14-24 years) to evaluate the acceptability of the ARORA app using a modified version of the User Version of the Mobile Application Rating Scale [30] and a subsequent focus group. We found that while the app generally performed well at promoting mindfulness and social awareness, it could benefit from being more centered around Hopi/Tewa culture and values in further development.
- We reflect on the process of conducting remote user testing with limited connectivity and technical resources, offering guidance for future work in rural and remote mHealth.

## Methods

### Ethical Considerations

Our study protocol was reviewed and approved by Northern Arizona University’s Institutional Review Board (number 1647272) and permitted by the Hopi/Tewa Cultural Preservation Office. Hopi/Tewa Cultural Preservation Office oversees and approves all research projects for and in collaboration with the Hopi tribe. All participants were recruited through community partnerships, and any participants younger than 18 years were required to receive explicit permission from their guardians before partaking in this study. All participants in this study underwent informed consent, with adult participants signing consent forms and minor participants signing minor assent forms with their parents or guardians. All participants of this study were compensated for their time and opinions with US $25 Amazon gift cards. Participant data in this work were anonymized and codified.

### Statement of Positionality

This research was led by investigators with backgrounds in computer science, mental health, and education. Of the 4 principal investigators (MVH, AA, DHJ, and AFC) directing this project, 1 was Hopi/Tewa. Additionally, our team included a community research coordinator from the Hopi/Tewa tribe who was responsible for recruiting participants and conducting the user testing sessions. While the remaining investigators (SH and MA) were not Hopi/Tewa themselves, they collectively hold over 2 decades' worth of experience collaborating with Indigenous communities in co-designing health interventions or technologies to meet community needs and goals. Additionally, our work was overseen by a community advisory board (CAB), consisting of 6 members of the Hopi/Tewa tribe.

### Development of Prototype

**Figure 1.**
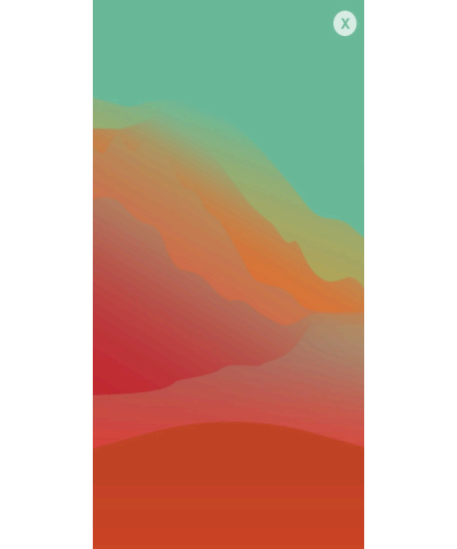
Mindful walking screen.

**Figure 2.**
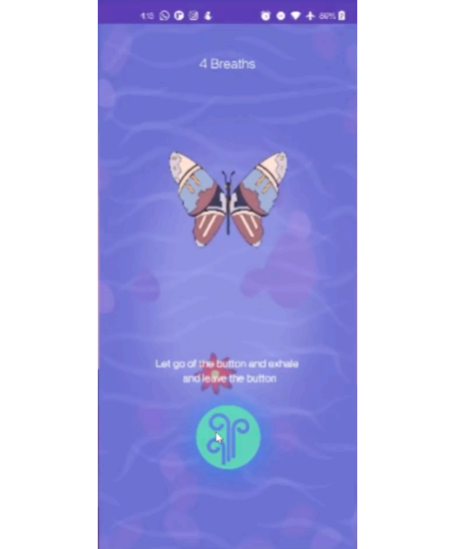
Mindfulness breathing screen.

The ARORA prototype was developed using Java and Kotlin for Android devices and emulators. This app included 3 mindfulness-oriented activities that were ideated in collaboration with our CAB members. Each activity combined Hopi/Tewa cultural values with evidence-based mindfulness research. The mindfulness activities included a mindful walking exercise (Figure 1), a mindful breathing exercise (Figure 2), and a mindful meditation exercise. Mindful walking has been shown to reduce the effects of depression, anxiety, stress, and burnout among individuals [31,32], which inspired our design of the walking activity. The exercise incorporated peaceful audio guides for the user to listen to as they walked and observed their surroundings. The mindful breathing activity, centered on guided breathing practices shown to reduce anxiety [33], included audio guides for taking in breaths, including imagery of feathers. The meditation activity offered a mini-game, in which users would listen to the guiding audio in the background as they lined up dots on the screen, requiring them to direct their attention to the app for the entire duration of the activity [34]. The audio scripts for the breathing, walking, and meditation exercises were written by a psychologist coauthor and then modified by our CAB members, ensuring that the resulting content was grounded in Hopi/Tewa culture and reflected existing practices of resilience.

**Figure 3.**
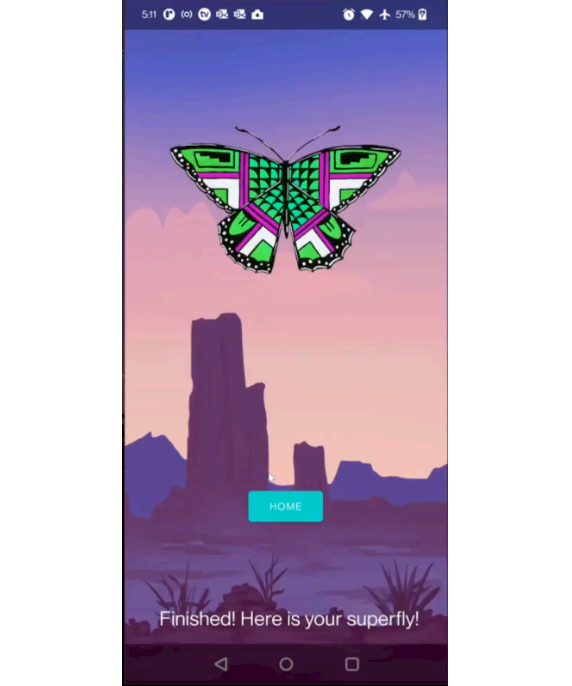
Users combine their butterflies to create a Superfly.

In addition to the mindfulness activities, the app included 2 augmented reality (AR) activities that were designed to encourage collaboration and community support among users. In each of these games, users were able to view and catch animated butterflies through AR, interactively moving their mobile devices and positioning their cameras to view animated butterflies in their real-world environments. One of the AR activities called “Superfly” was collaborative, requiring players to work synchronously with one another to collect butterflies through the app and then combine their butterflies together to create a Superfly (Figure 3). The Superfly exhibited colors from each of the butterflies provided by the players, rewarding them for their collaboration. The goal of Superfly was to encourage positive and collaborative interactions between users to further build a sense of community with one another. This design choice reflects prior mHealth work with Indigenous populations, which tie community values and practices for establishing wellness and resilience, such as positive psychology [35-38]. This activity was co-designed and planned in collaboration with our community partners.

**Figure 4.**
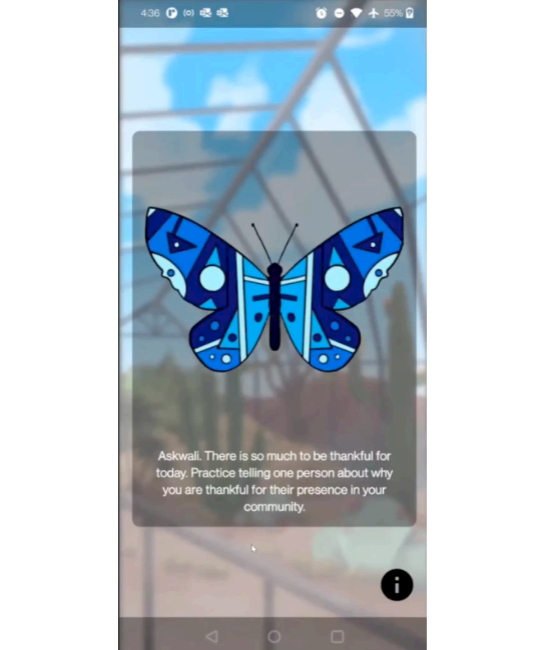
Lesson associated with a caught butterfly.

While the goal of Superfly was to promote collaboration and a sense of community, users could also capture butterflies on their own, viewing them in a virtual atrium. The virtual atrium allows users to view each of the butterflies they have caught already. Each butterfly caught had an associated, culturally centered lesson that users could view within the atrium (Figure 4).

### Adapting the Research Protocol in Alignment With PAR Practices

This work was conducted between May 2021 and August 2021, at a time when COVID-19 restrictions were omnipresent, but especially important among tribal communities. AI/AN communities were disproportionately impacted by the pandemic [39], necessitating higher precautions for holding meetings. Consequently, all user testing initially planned to be held in-person was moved online. Originally, all participants were to test ARORA on Android devices on Northern Arizona University’s campus, where the research team could directly view their actions within the app. Since these studies took place over Zoom, we instead preloaded ARORA onto Android emulators running from the researcher’s computers. Members of the research team screen-shared these emulators over Zoom, granting the participants remote access, such that they would be able to engage with the app using their computer mouse. This adaptation to the protocol required that participants engaging in this study have access not only to the internet but also to a computer.

AR-based activities, such as Superfly, were modified to no longer require cameras, instead showing the butterfly animations over a static screen. While this limited the usability testing of the AR features, offering a modified version of Superfly still allowed us to receive valuable feedback on the acceptability of the activity and areas for improvement in its design. The walking activity could not reasonably be evaluated without physical devices, so it was omitted from this study.

Maintaining a flexible protocol by following PAR practices supported our research team in establishing trust with our community partners—iteratively adapting the prototype to meet their needs and desires. By not adhering to typical research timelines or rigid requirements, this practice also supported us in establishing trust. Ultimately, the goal of this work was to develop technologies that support the needs of our community partners and responding to needs through adaptation was essential.

### Participant Recruitment and Demographics

Collaborating with our community partners, we recruited 9 participants, each of whom was Hopi/Tewa. We followed a similar process for recruitment as we did for our 2019 focus groups [26]: we found participants through community messages that our CAB partners released and used snowball sampling, thereby recruiting members through word of mouth. All participants were considered “youth” if they were between the ages of 16 and 24 years. Of the 9 participants, 7 identified as having grown up in “completely rural” or “mostly rural” communities.

### Focus Groups and Usability Testing

Between February and July of 2021, we held 3 focus group sessions, which were each approximately 60 minutes long, with 2-3 participants attending each session (9 participants total). These sessions focused on evaluating the usability of ARORA, also asking participants for feedback on how the app could be improved to better serve Hopi/Tewa youth. Again, while we initially planned to hold these sessions in-person, we modified our plans to hold them online due to restrictions brought on by the COVID-19 pandemic. All sessions began with the participants and researchers in 1 Zoom room. In this collective Zoom room, researchers debriefed participants in what they would be doing for the remainder of the session, obtained consent, and answered questions as needed. Participants were also asked to complete a brief background survey after joining the Zoom room, prior to testing the app.

Then, each participant was placed in a breakout room with at least 2 researchers, where they were asked to use the app. One researcher would act as a facilitator, providing the participant with instructions on what to do within the app, while the other would take notes and ask the participant questions as needed. To accommodate for focus groups being held over Zoom, 1 researcher in each breakout room ran the app from an Android emulator on their computer and shared their screens with and granted remote access to a participant through Zoom. This allowed participants to still experience the app and provide useful and important feedback on its design. We continued holding focus groups until no new themes emerged from the focus group responses [40]. Each participant was given several tasks to complete within the ARORA app. They were encouraged to complete the tasks with no guidance but could ask for help from their facilitators when needed. Our goal was to understand how easy or difficult completing each of the tasks was without assistance. The tasks participants were asked to complete are listed in Table 1.

**Table 1.** Instructions for usability testing.

Task, n	Instructions
1	Log into the app with given login credentials.
2	Complete the mood and stress survey.
3	Find the breathing learning exercise and complete it for 5 breaths.
4	Find the meditation learning exercise, select any of the available themes, and complete it for 90 seconds.
5	Find the atrium.
6	Find the blue butterfly in the atrium.
7	Read out loud the lesson associated with the blue butterfly.
8	Find the pollen points and read out loud how many points the account has.
9	Find the butterfly catching game and play it for 1 round.
10	Review how many of each color of butterflies are there in the atrium.

After testing the app, participants were asked about their experiences with and opinions toward the app. They were also asked to answer a series of open-ended questions: (1) What did you like about the app? (2) What are the things that you did not like about the app? (3) What would you change or add?

#### Thematic Analysis of Focus Group Transcripts

We conducted a grounded theory analysis on the transcripts from focus groups [41]. After the first focus group, the first author (SH) would apply open codes to responses to focus group questions and in a second pass, the first and second authors (SH and MVH) would meet to discuss and refine open codes using a consensus approach. The 2 authors then collaboratively defined axial themes that emerged to encompass each of the open codes. For the 2 subsequent focus groups, the 2 authors repeated this procedure, using a constant comparison approach to applying codes and themes to subsequent transcripts and expanding our codebook and themes for the second focus group; by the third focus group, we did not add new codes or themes. We report on the axial themes, open codes, and examples in Table 2. Emergent themes included graphics, activities and features, usability, impact, and culture.

**Table 2.** Qualitative results from focus group sessions.

Open codes	Axial codes	Quotes
Graphics	Hopi-specific graphics Meaningful or visually appealing colors	“I think it would be interesting or more valuable to highlight like the landscapes unique to the Hopi/Tewa community like the Mesa.” “[The butterfly] kind of helped establish that connection between the community and the audience, because of how it was, like, incorporated. The Native American design is there and seeing that image, it made me think of artists from within that community who use those types of designs and that style of art, so that was great.” “I really appreciated the...tie to the community as well through the background images in that they were representing the desert landscape.” “When you open an app, the visuals is the first thing that’s gonna catch your eye, so the colors were really nice.” “I thought that the layout was pretty good and the colors in the app were nice.”
Activities and features	Mood and stress survey is beneficial Want more mindfulness-focused features Want a feature for users in crisis	“[I liked] the little journal inputs at the beginning like the daily [survey]...I feel like that could really help you.” “I [would] put like affirmations kinds of things, like daily quotes...things that could make you think and really give you some good thoughts to start off your day because...that’s always good...and just things that would help you.” “Maybe...in certain situations, like if someone is in the middle of...[a] panic attack or...something that could help with that...would be really cool.” “It was nice how you were able to put how you are feeling today and I would...like adding something to log how you feel each day so that way you or someone else can go back to how they were feeling.”
Usability	Tutorial/walkthrough for new users Guidance for activities Guidance on where to find specific information	"It did take me a while to kind of familiarize myself with the kind of tabs at the bottom...But once I did familiarize myself, I believe I could find those where I’m needing to go again, easier.” “I wish that there was...more information included in some sections. I know for some areas, like the atrium portion, I wasn’t sure of how to determine...what those butterflies were for and how I could use them.” “I have noticed in some previous apps they show you some features in the beginning, like kind of an intro.”
Impact	Some youth struggle with vocalizing feelings Having an app readily available could help youth manage stress and other negative emotions	“I think this is really needed. I think...a lot of people my age are scared...you know...they don’t want to confide or maybe talk to people about it. If they were able to do it within an app or something like that can actually help them.” “I don’t like to confide in anybody, because I don’t know—there’s some things I can confide in people about but not all the time, so I think if an app would be a good way to help people— if its stress or if it’s anxiety or anything like that it would be good because everyone is on their phone all the time. So if they have a problem and don’t feel like talking about it they can just pull out their phone and go ‘oh I have this app, I’m gonna use this.’ It would be good help.”
Culture	Including Hopi/Tewa language Hopi-specific, rather than a general mental health app	“I like how you guys added ‘Askwali’ in there, meaning ‘Thank you’ in Hopi/Tewa. I thought that was really good.” “I know there’s some other [mental health apps] out there and I like the idea that this app has Hopi—includes Hopi/Tewa in there. And that’s what I really like about it and why it’s different.”

#### Demographics

In the background survey, participants were asked to answer basic demographic questions about themselves, specifying their gender, age, race, and tribal affiliation. Each of the 9 participants was Hopi, with 3 indicating that they had additional tribal affiliation as well. Participant ages ranged between 16 and 24 years. Additionally, we asked participants to specify the types of settings in which they grew up in: “completely rural,” “mostly rural,” “suburban,” “mostly urban,” and “completely urban.” The results of the background survey indicated that the majority of participants (7 out of 9) grew up in rural areas, highlighting the importance of considering rurality in co-designing software for the Hopi/Tewa population. In addition to asking demographic questions, we also asked participants about the types of technology they had access to and the locations in which they lived. Seven of the 9 participants reported using smartphones on a daily basis, with the remaining participants using smartphones only once or twice per month. The mean average age of participants was 19.8 (SD 2.619) years; 8 of the 9 (89%) participants were female.

## Results

### Principal Findings

One of the most prevalent themes in our findings was a need and desire for the app to be more specific to the Hopi/Tewa community. The version of the ARORA prototype used in these focus groups was co-designed with both Hopi/Tewa and Navajo community partners and iterated on following feedback received from a focus group held in 2019. While the app was modified after the initial 2019 focus group to emphasize and reflect Hopi/Tewa culture and values, the results from this 2021 set of focus groups show that there was room for improvement in terms of its cultural responsiveness. Several participants provided specific suggestions for how to modify the app so that it would be more grounded in Hopi/Tewa culture. For example, the graphics in the app could be more Hopi-specific, with one participant stating, “I think it would be interesting or more valuable to highlight…landscapes unique to the Hopi/Tewa community like the Mesa.”

Another area for improvement was usability. Several participants had difficulty finding components of the app, such as the atrium and the mindfulness exercises, at times needing help from the facilitators. Finding these components of the app also required more time from some participants, due to confusion on where they were located. One participant explained their confusion with the atrium, stating, “I wish that there was...more information included in some sections. I know for some areas, like the atrium portion, I wasn’t sure of how to determine...what those butterflies were for and how I could use them.” To help clarify to users how to use the app, one participant suggested that we develop an introductory tutorial, stating “I have noticed in some previous apps they show you some features in the beginning, like kind of an intro.” A tutorial for new users to the app could limit the amount of time that they would need to take to familiarize themselves with its features, showing them information on what activities are available and where they can be found. While the size of this study was small, the resulting feedback was still valuable in informing the iterative design of a culturally grounded mHealth app. The goal of this study was to identify perceptions toward the prototype and areas for improvement to ensure that it effectively tied into Hopi/Tewa culture while promoting resilience.

### Quantitative Results on Perceptions of ARORA

Following testing the ARORA prototype, participants were asked a series of questions surveying their perceptions of the app, each of which they were asked to answer on a scale of 1-5, 1 meaning “highly disagree” and 5 meaning “highly agree.”

The results of the survey imply neutral-to-positive reception, with all scores falling at or above the average score of 3.0, which was neutral. Additionally, we asked participants to rate the app on a scale of 1-5 (1 being “very little” and 5 being “very much”) for its cultural responsiveness, social awareness motivation, mentorship motivation, competition motivation, and creation motivation. Multimedia Appendix 1 shows the average values among the responses.

Indeed, the results from these surveys suggest that the app must be adapted to have higher Hopi/Tewa cultural centeredness, reflected by the results’ lowest response score. Additionally, further evaluation is necessary for determining the psychological impacts of this app.

## Discussion

### User Testing Results, Feedback, and Considerations for Further Development

The majority of our participants lived in a mostly rural or completely rural environment when they were growing up. This is very important to acknowledge, as rurality itself can be a critical factor in technology design—particularly as it intersects with pervasive broadband connectivity [42]. This adds a layer of complexity to the ARORA app, as it must be able to operate correctly when the internet connection is unavailable or unstable, a concern that was highlighted during these usability sessions when high network latency influenced usability. Indeed, challenges with intermittent connectivity have impacted prior mHealth work in rural spaces [24,29], presenting a barrier to telehealth. This points to a growing need in the mHealth space to develop connectivity-aware technologies that can still support individuals when internet access is limited. Reiterating reflections of Wyche et al [24] working with rural populations, mHealth technologies must be informed by community affordances.

User experiences with ARORA during intermittent connectivity and lagging reflect design theories grounded in consistent evaluations, such as Formative Situations [28]. Following this design practice, we considered the unique challenges present in using the ARORA prototype across common, lived experiences of stakeholders to understand areas for improvement. This practice is beneficial for the mHealth space, as it supports researchers and practitioners in receiving early feedback for continuous and iterative development with the goal of designing technology that meets the needs of their target audience.

**Figure 5.**
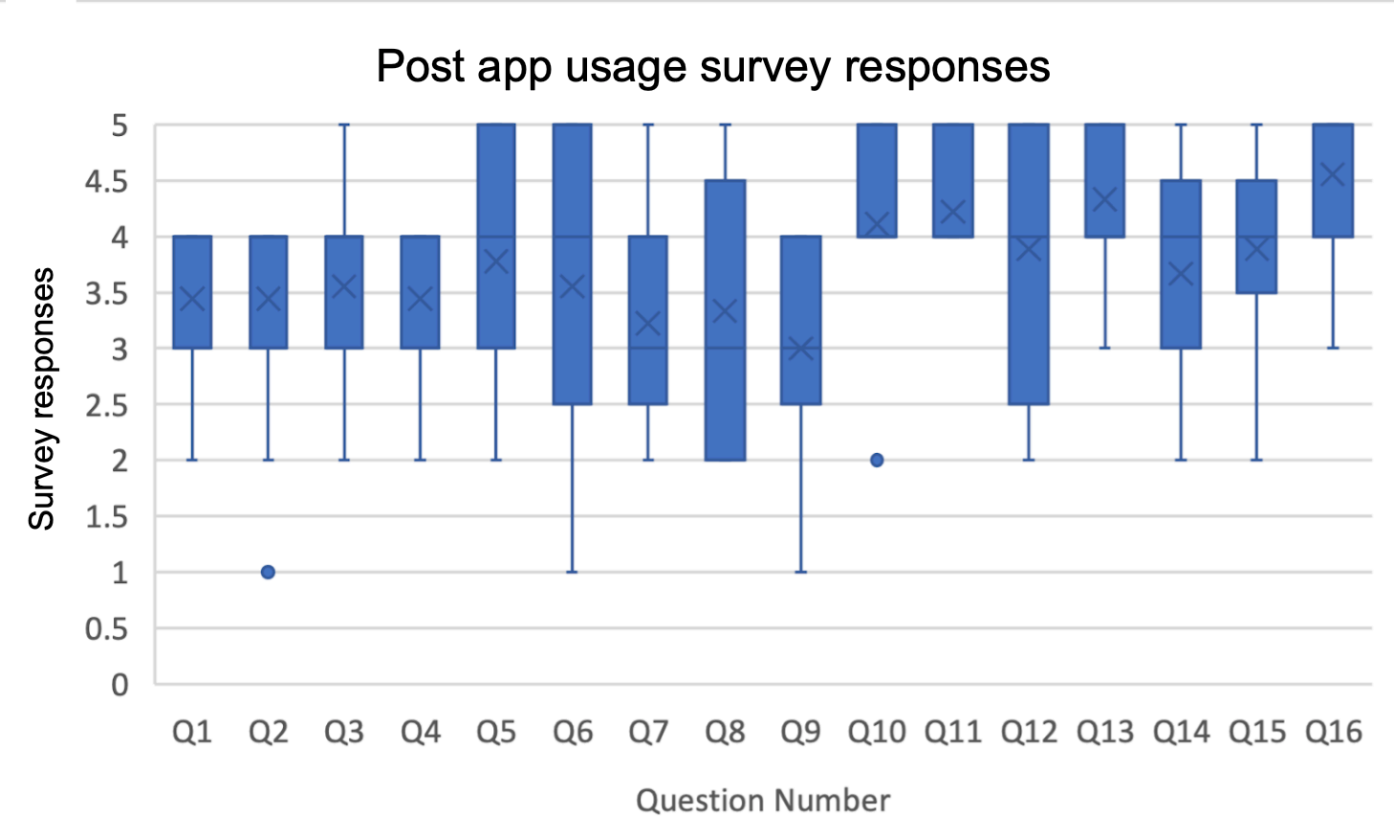
Survey responses.

Several participants emphasized a desire for the app to be more grounded in Hopi/Tewa culture. The need for ARORA to tie more closely to Hopi/Tewa lifeways is also underscored by the survey results shown in Figure 5. For instance, the average score for “This app used images that made me feel connected to my Hopi/Tewa culture” was 3.55, indicating predominantly neutral responses. This reflects ongoing calls in the human-computer interaction space to increase cultural and identity relevance in mHealth technologies [20]. At the time of writing, we have entered a formal partnership with Hopi/Tewa Tribe Behavioral Health Services to further refine the ARORA app and to address feedback from focus group sessions described in this paper. Reviewing the results from this study, we find that ARORA can be improved by increased Hopi-specific aesthetics and text-based content, being more specifically centered around a single tribal community. Future usability testing will benefit from the app being installed on physical devices (eg, testing on smartphones, rather than emulators), with in-person collaboration to fully test all activities within the app, such as the mindful walking activity.

While feedback toward ARORA’s concept and purpose was valuable for its iterative design, the results of this study do not depict its impacts on mental health. The survey questions presented in Table 3 dominantly evaluated cultural alignment, with some centering on mindfulness concepts. Future work should involve more rigorous evaluation of mindfulness and mental health concepts through randomized clinical trials. Additionally, evaluating the mental health effects of this app will require a larger participant pool, as this study involved only 9 members of the Hopi/Tewa community.

**Table 3.** Survey responses, with mean scores and standard deviation.

Survey item	Scores, mean (SD)	
If given the chance, I want to use this app again.	4.55 (0.726)	
Being able to participate in more individual-based competitions and activities would encourage me to use the app more often.	4.33 (0.707)	
Being able to share my activities with peers would encourage me to use the app more often.	4.22 (0.972)	
Learning about how my peers use this app would encourage me to use the app more often.	4.11 (0.928)	
Being able to communicate and receive feedback about my activities from trusted mentors in my community would encourage me to use the app more often.	3.88 (1.270)	
I am likely to recommend this app to others like me.	3.88 (0.928)	
This app supports Hopi/Tewa ways of being a strong person who can deal with tough times.	3.77 (1.093)	
Being able to contribute something of my own creation (eg, videos, photos, sound clips, and artwork) to the app experience would encourage me to use the app more often.	3.66 (1.0)	
This app used images that made me feel connected to my Hopi/Tewa culture.	3.55 (1.424)	
This app increases my knowledge of how to practice being aware of my physical surroundings.	3.55 (0.882)	
Using this app is likely to increase my awareness of my surroundings.	3.44 (1.130)	
Using this app is likely to increase my awareness of my feelings.	3.44 (1.130)	
This app increases my knowledge of how to practice being aware of my feelings.	3.44 (0.726)	
This app supported spiritual mindfulness to Hopi/Tewa ways of being.	3.33 (1.225)	
This app used language/text/terms that made me feel connected to my Hopi/Tewa culture.	3.22 (0.972)	
As a Hopi/Tewa community member, this app reflected me and my culture.	3.0 (1.0)	

### Adapting Research Protocols With Hard-to-Reach Populations to Accommodate for Remote Research

Future work in mHealth, especially work done with rural communities, must consider events that hinder in-person or synchronous evaluations, conceptualizing potential ways to digitize practices in support of limited affordances. Several factors can influence the ability to conduct research with remote communities, from infrastructural barriers to local government policies to geographic limitations. However, developing adaptable protocols can support future work in continuing progress with their partnered communities, in spite of limitations beyond their control. In this work, we were required to remove certain activities from the evaluation protocol that simply could not be adapted with the affordances available at that time. This practice of adaptation is useful for other hard-to-reach populations, such as stakeholders with lowered immune systems. Future research in mHealth can allow for this flexibility by adopting PAR frameworks and practices, emphasizing modifications to planned work as necessary.

### Limitations

Due to the nature of our usability sessions occurring during the COVID-19 pandemic, we experienced limitations in how testing of the app was carried out. We needed to shift away from individual and group meetings to meet remotely so that we could prioritize the health of participants and researchers. This meant that we had participants use the app via an emulator, which was run on the researcher’s computers so that participants would have remote screen access. Participants predominantly joined the Zoom meeting from Hopi, where internet connectivity tends to have a higher latency. Moreover, the emulator itself added delays in processing time. As a result, the app tended to lag by a few seconds whenever screens were swiped or buttons were clicked. This made completing certain tasks within the app, such as the meditation activity, difficult for participants.

This work is an example of challenges present when working with rural populations. Although evaluating digital artifacts in person is ideal for understanding user experiences and perspectives, it is not always feasible. The ability to hold in-person meetings and testing sessions hinges on geographical, infrastructural, and bureaucratic affordances. This work took place during the COVID-19 pandemic, which AI/AN communities were disproportionately affected by. Consequently, many tribal populations, including Hopi/Tewa, restricted group gatherings, especially including those external from their communities. There was still a need and interest for our work to continue, reflected by conversations with our community partners, which required that we adapt our protocol (including limiting the testing of movement-based activities).

The sample size may be considered a limitation of our study. It is important to consider that sample sizes are not unusual in qualitative research, and that of Hopi/Tewa’s population is small (with approximately 7791 people living on Hopi, accounting for half of the tribe’s membership). Additionally, our findings highlighted themes that emerged across focus groups. While this work could have benefitted from a larger sample size with a broader range of Hopi/Tewa participants (specifically regarding gender identity), the findings are still valuable in informing the ongoing and iterative design of an mHealth app. This process follows the PAR practice of Formative Situations, as defined by Robinson et al [28], which supports evaluating artifacts across contexts that stakeholders may experience (such as intermittent connectivity).

### Conclusions

In this work, we conducted user studies on the initial prototype of a mHealth smartphone app, ARORA, designed for supporting emotional resilience among Hopi/Tewa youth. The goal of this study was to gather feedback from stakeholders as part of a larger, iterative design process, aligning with the principles of PAR. We faced challenges conducting this work due to the COVID-19 pandemic. Due to the flexibility of our protocol, we adapted our user study plans to accommodate remote evaluations. As a result, we developed a better understanding of important infrastructural limitations, such as intermittent internet connectivity, to inform our prototype’s iterative design. Additionally, we received qualitative feedback on this initial prototype through focus groups, after reaching thematic saturation. The findings from this work highlight a need for increased cultural responsiveness within ARORA in further iterations of its design. Participants noted value in the existing Hopi/Tewa language incorporated in the app but concluded that Hopi-inspired aesthetics and practices could further support it in being culturally relevant.

## Supplementary material

10.2196/77898 Multimedia Appendix 1 Mindfulness survey responses.

## Abbreviations

AI/AN: American Indian/Alaska Native
AR: augmented reality
ARORA: Amplifying Resilience Over Restricted Internet Access
CAB: community advisory board
mHealth: mobile health
PAR: participatory action research
