# Supplementary material for: User Testing an mHealth Behavioral Health App for Hopi/Tewa Youth During the COVID-19 Pandemic: Usability Study
Source: JMIR Form Res. 2026 Mar 24;10:e77898. doi: 10.2196/77898 (PMC13012818; doi:10.2196/77898)
Supplement: Multimedia Appendix 1 [file formative-v10-e77898-s001.docx]

Multimedia Appendix 1

Table S1. Mindfulness survey responses.

| **Mindfulness question topic** | **Average response** |
| --- | --- |
| Creative motivation | 3.667 |
| Competitive motivation | 4.333 |
| Mentorship motivation | 3.889 |
| Social awareness motivation | 4.167 |
| Cultural responsiveness | 3.389 |
| Mindfulness | 4.472 |
